# Neutrophil Integrins and Matrix Ligands and NET Release

**DOI:** 10.3389/fimmu.2016.00363

**Published:** 2016-09-19

**Authors:** Xian M. O’Brien, Jonathan S. Reichner

**Affiliations:** ^1^Division of Surgical Research, Department of Surgery, Rhode Island Hospital, Providence, RI, USA; ^2^Warren Alpert Medical School, Brown University, Providence, RI, USA

**Keywords:** NETs, extracellular matrix, neutrophils, integrins, Candida

## Abstract

Neutrophils are motile and responsive to tissue injury and infection. As neutrophils emigrate from the bloodstream and migrate toward a site of affliction, they encounter the tissue extracellular matrix (ECM) and thereby engage integrins. Our laboratory studies the neutrophilic response to the fungal pathogen *Candida albicans* either in the filamentous state of the microbe or to the purified pathogen-associated molecular pattern, β-glucan. We have gained an appreciation for the role of integrins in regulating the neutrophil anti-Candida response and how the presence or absence of ECM can drive experimental outcome. The β2 integrin CR3 (complement receptor 3; αMβ2; Mac-1; CD11b/CD18) plays an important role in fungal recognition by its ability to bind β-glucan at a unique lectin-like domain. The presence of ECM differentially regulates essential neutrophil anti-fungal functions, including chemotaxis, respiratory burst, homotypic aggregation, and the release of neutrophil extracellular traps (NETs). We have shown that NET release to *C. albicans* hyphae or immobilized β-glucan occurs rapidly and without the requirement for respiratory burst on ECM. This is in contrast to the more frequently reported mechanisms of NETosis to other pathogens without the context of ECM, which occur after a prolonged lag period and require respiratory burst. As expected for an ECM-dependent phenotype, NETosis and other neutrophil functions are dependent on specific integrins. The focus of this review is the role of ECM ligation by neutrophil integrins as it pertains to host defense functions with an emphasis on lessons we have learned studying the anti-Candida response of human neutrophils.

## Prevalence and Risk Factors for Candidiasis

*Candida albicans* exists as normal flora of the skin and GI tract but can become a serious and life-threatening infection. Candidiasis can present either locally as mucocutaneous infection or as the more severe invasive form of the disease. Predisposing factors lending to loss of host control of the colonized organism are likely to be a combination of host as well as microbial factors (1). Invasive candidiasis continues to be a significant medical problem and Candida ranks as the fourth leading pathogen in causing nosocomial infection with mortality up to 40% in spite of available anti-fungal therapy (2). Infection can take place in any bodily organ and systemic infection can involve coincident infection of multiple organs, as well as the blood.

Clinical risk factors for acquisition of Candida infection include neutropenia or a neutrophil defect whether heritable or epigenetic, systemic antibiotic usage, central venous catheter, mucosal damage, and prolonged stay in the ICU even in the presence of surfeit neutrophils (3, 4). Candida infection is remarkably high in non-trauma emergency surgical patients with a prolonged ICU stay, reaching a rate of 21.7/100 discharges, higher than other established high-risk populations (5, 6).

## Fungal Recognition

*Candida albicans* is a polymorphic fungal pathogen that can grow as yeast, pseudohyphae, and true hyphae and the ability to switch between phenotypic states is an essential virulence factor complicating immune detection (7–9). Neutrophils respond to infectious fungi in a variety of ways, including phagocytosis, production of reactive oxygen species (ROS), degranulation, recruitment of other leukocytes, and the more recently recognized release of neutrophil extracellular traps (NETs). In its budding yeast form, *C. albicans* is small enough for neutrophils to phagocytose. This response involves uptake of microbes into the phagosome, where fusion of cytotoxic granules and oxidative products facilitate microbial killing (10). The invasive filamentous forms of *C. albicans* are too large to be engulfed, necessitating other cellular strategies for anti-fungal response and clearance (11–16). The recently described process of NETosis, where NETs consisting primarily of DNA studded with histones and components of cytotoxic granules are extruded into the extracellular space, accomplishes the dual functions of both immobilizing and killing harmful microbes where phagocytosis is not feasible (17).

Innate immune cells recognize *C. albicans* by binding to molecules present in the fungal cell wall. β-glucans are a class of long-chain polymers of glucose in β-(1,3) (1,6)-linkages that are conserved in microbial structures but not found in mammalian cells and, thus, are considered a pathogen-associated molecular pattern (PAMP) (11, 18, 19). Pattern recognition receptors (PRRs) on cells of the innate immune system discern PAMPs as being non-self and initiate antimicrobial host defense mechanisms through activation of intracellular signaling pathways. With regard to recognition of β-glucan, two receptors have received the most attention; the integrin CR3 and the C-type lectin Dectin-1 that may exert non-overlapping roles in clinical and experimental host defense. To parse the relative roles of these receptors, one must take into account the species of the host and immune cell type being studied as the anti-fungal role of these receptors can differ between monocyte/macrophages and neutrophils. Differences may also lie in the specific immune function being assayed and the morphological form of the Candida.

Dectin-1 plays a key role in *C. albicans* control in mice such that mice defective in Dectin-1 are susceptible to fungal infections while CR3 knockout mice are more resistant to challenge with disseminated *C. albicans*, suggesting that CR3 has a non-protective, or suppressive effect on murine host defense (20). In humans, Dectin-1 has been shown to be important in control of mucocutaneous but not systemic infection (21, 22). This was supported by a study of a family with a mutation of caspase recruitment domain-containing protein 9 (CARD9), a signaling molecule downstream of Dectin-1 (23). In this family, the CARD9 defect presented as a predisposition to mucocutaneous candidiasis similar to the absence of Dectin-1, mediated by a cytokine production defect of monocytes and macrophages (21, 23). Neutrophils from leukocyte adhesion deficiency (LAD) type 1 patients that are devoid of CD11b/CD18 but which express Dectin-1 failed to internalize *Saccharomyces cerevisiae* or unopsonized zymosan demonstrating the primacy of CR3 in phagocytosis of unicellular yeast and β-glucan-containing particles (24). In short, phagocytosis of unopsonized yeast or β-glucan-containing particles is primarily mediated by CR3 in human phagocytes and by Dectin-1 in murine cells (25). It is not clear why the genetic absence of CR3 has such different implications for anti-fungal immunity in mice and humans. This is often correlated with the notion that CR3 ligation by β-glucan particles fails to induce respiratory burst thereby limiting this host defense mechanism (26). However, we and others have shown that human neutrophils induce a CR3-dependent respiratory burst to fungal hyphae or immobilized purified β-glucan as a model of the response to non-phagocytosable filaments (11, 12, 15). Given the multifaceted role of CR3 in immune response to a pathogen, it is difficult to ascribe a mechanism to the increased resistance in CR3 knockout mice.

With regard to recognition of non-phagocytosable fungal hyphae, our laboratory showed that antibody blockade of either fungal cell wall β-glucan or neutrophil CR3 was sufficient to obviate the respiratory burst of human neutrophils; antibody blockage of Dectin-1 had no effect (11, 13). In addition to the host defense mechanisms affected by CR3 and Dectin-1 individually, there is solid evidence for a crosstalk pathway connecting these PRRs. Li et al. showed a mechanism dependent on the RhoGTPase exchange factor, Vav, through which binding of β-glucan to Dectin-1 resulted in CR3 activation in both murine and human cells (27). This highlights the potential complexity of working toward a more complete understanding of the differential nature of immune recognition of *C. albicans* hyphae and yeast forms. A significant step forward in this regard is found in a report by Lowman et al. (22) in which a novel cyclical, or “closed chain” structure of β-glucan was found in *C. albicans* hyphae but not in yeast. These authors purified β-glucan from *C. albicans* yeast and hyphae into water-insoluble microparticulate form and showed that the β-glucan extracted from hyphae, but not yeast, produced a potent IL-1 response by human monocytes and macrophages, which was Dectin-1-dependent. Whether monocyte Dectin-1 can recognize cyclical hyphal β-glucan within the cell wall of the organism remains to be seen. Findings to date suggest that Dectin-1 recognition of Candida hyphae is limited to bud scars where β-glucan is particularly exposed, it does not appear to recognize β-glucan along hyphal filaments (28). Whether neutrophils exhibit differential responsiveness to these β-glucan isoforms has not yet been determined. Therefore, the differential responsiveness of innate immune cells to the yeast and hyphal forms of *C. albicans* may well be due to variance in the structure of the prominent fungal PAMP β-glucan. Work from our laboratory and others show that CR3 is most likely the prominent immune receptor on human neutrophils and is able to detect β-glucan within fungal filaments (11, 13, 15, 29). As CR3 serves as both a PRR and an extracellular matrix (ECM)-binding integrin, it plays a critical role in integrating tissue environment and microbial recognition, driving neutrophil anti-fungal immunity.

## Role of Integrins in Anti-Fungal Immunity

All cell–cell and cell–ECM adhesive events occur extracellularly but are translated into cellular responses by communication across the plasma membrane through the action of integrins (30). Integrins are essential for proper regulation of a number of fundamental physiological processes, including tissue morphogenesis, inflammation, immune responsiveness, wound healing, and regulation of cell growth and differentiation. All cells express a contingent of integrins and respond to integrin activation by cytoskeletal-dependent processes, such as shape change, adhesion, spreading, migration, and/or phagocytosis (31). Among 24 αβ heterodimers that have been reported in vertebrates, the β2 family (αLβ2, αMβ2, αXβ2, and αDβ2) are specifically expressed on leukocytes (31). Leukocyte β2 integrins regulate many aspects of immune or inflammatory responses because, unlike cells that reside within solid tissues, circulating leukocytes by necessity relocate during the course of immune reactions. In so doing, they dynamically adhere and de-adhere to cells of the vasculature, to other immune cells, and to components of the ECM, in order to ultimately contact the foreign body or pathogen at the site of infection or injury. Evidence for the physiological significance of leukocyte integrins is highlighted by the recurrent, life-threatening infectious episodes observed in LAD patients that are genetically deficient for expression of β2 integrins (32). In stark contrast to impaired host defense found in the absence of β2 integrins, sustained and improper activation of these integrins contributes to the pathogenesis of autoimmune diseases, chronic inflammatory disorders, and ischemic stroke (33).

Complement receptor 3 (CR3; αMβ2; CD11b/CD18; Mac-1) is a member of the β2 integrin family, yet it functions like no other integrin and, in some ways, like no other receptor yet described in nature. In general, receptors can be defined as having a canonical ligand that binds with characteristic affinity to a single binding site which, in turn, leads to a characteristic intracellular response. In stark contrast, CR3 has two spatially distinct binding sites, the so-called I-domain and the lectin-like domain, that bind completely different ligands and results in differing cellular responses. The I-domain itself is a highly promiscuous binding site with over 30 structurally unrelated ligands shown to be capable of binding at that domain alone, including iC3b, fibrinogen, ICAM-1, fibronectin, heparan sulfate, and factor X (34, 35). I-domain ligands are both host- and microbial-derived such that a multitude of immune effector functions executed by inflammatory neutrophils are entirely mediated, or regulated, by CR3. As with other integrins, ligand binding is regulated by the structural state of activation such that when in a bent conformation the integrin is in a low-affinity state that is modulated upon activating signals that can originate internally (inside-out) or externally (outside-in). In either case, the receptor assumes an upright conformation consistent with high-affinity ligand binding that can be further regulated by receptor clustering resulting in avidity modulation (36). The lectin-like domain is spatially distinct from the I-domain, and is noted for its ability to bind the glucose polymer β-glucan (11, 18, 37–39). Ligation of purified fungal β-glucan to CR3 is sufficient to induce a signaling response (39). The ability of CR3 to mediate neutrophil recognition of fungi and initiate signaling identifies it as the only integrin that also serves as a PRR.

A novel aspect of CR3 bioactivity is that the manner in which it is ligated at its two binding domains has a profound effect on cellular responsiveness. This concept was first posited where Vetvicka et al. reported that murine and human natural killer cells could acquire cytotoxic capability for resistant tumor cells if targets were opsonized with iC3b, a well-described CR3 I-domain ligand, and effector cells were exposed to β-glucan, but not by either ligand alone (40–42). This increased cytotoxic activity could be inhibited with CR3-specific antibodies. Administration of β-glucan enhanced the activity of complement-fixing, anti-tumor antibodies *in vivo*, causing tumor regression and increased survival as compared to mice receiving either antibody or β-glucan alone (43–45). Surprisingly, this adjuvant activity of β-glucan in reducing tumor burden was shown to be mediated by neutrophils and did not occur in mice lacking either CR3 or complement or in mice depleted of neutrophils.

## Role of Extracellular Matrix in Anti-Fungal Immunity

**Figure 1:**
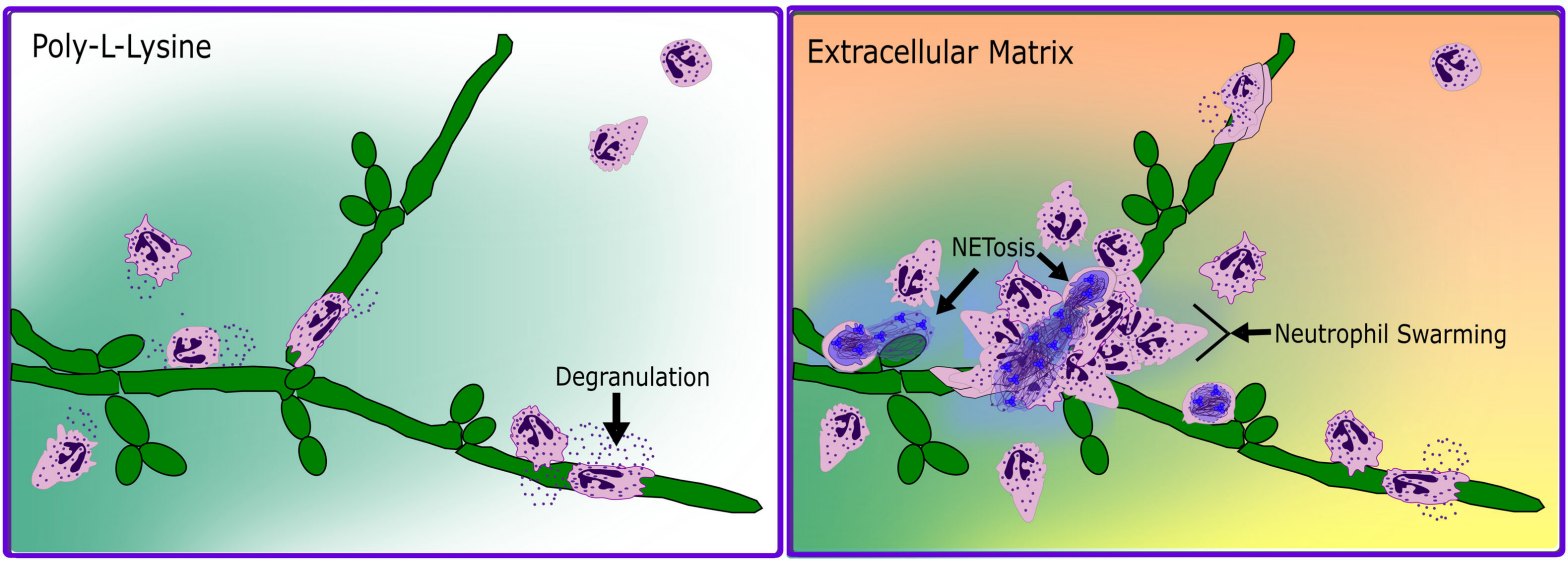
**Schematized neutrophil response to *C. albicans* hyphae in the absence and presence of extracellular matrix**. In the absence of ECM (poly-l-lysine, left panel), neutrophils respond to hyphae by chemotaxis, by degranulation and respiratory burst, and by wrapping around fungal filaments in a form of frustrated phagocytosis. In the presence of ECM (Extracellular Matrix, right panel), neutrophils chemotaxis to fungal filaments is faster and more directed, with degranulation and respiratory burst being actively suppressed until the multifocal contact of frustrated phagocytosis. Additionally, in a subset of cells contacting the fungal hyphae, a rapid, respiratory burst independent NETotic response is induced and followed by neutrophil swarming.

In a seminal paper by Carl Nathan in 1989, the respiratory burst of human neutrophils to soluble proinflammatory mediators was shown to be adhesion dependent and require attachment to either ECM components or human umbilical vein endothelial cells (46, 47). This adhesion dependence of neutrophil effector function provided an early the basis for suggesting the coordination of integrin and non-integrin stimuli to drive host defense in tissues. As CR3 mediates cellular interactions with ECM, and since all neutrophilic responses to tissue infections necessitate ECM contact, we focused our attention on investigating the role of CR3 as a fungal PRR via the lectin-like domain in the presence of fibronectin, a ubiquitous ECM molecule and I-domain ligand. We showed that the effect of CR3 on the anti-fungal response of human neutrophils to Candida is not a straightforward consequence of receptor ligation, but is directed by how it is ligated (11–13, 39, 48–52). We have found that upon dual ligation of CR3 by fibronectin and β-glucan, neutrophils demonstrated enhanced chemotaxis, swarming and aggregation, NETosis, and an actively suppressed respiratory burst (Figure 1). Antibody-blocking studies were used to show coincident ligation of CR3 at both the I-domain with the ECM component fibronectin and the lectin-like domain control neutrophil effector functions differently than ligation of either site alone (11–13, 39, 49–52). These studies additionally identified a CR3-mediated regulation of β1 integrins, driving a shift in fibronectin binding from α5β1 to α3β1 (12, 50, 52).

In order to be virulent, *C. albicans* must be capable of transitioning between yeast and hyphal forms (53). The yeast form is readily cleared by neutrophil phagocytosis, the mechanisms of which have been studied extensively. Less work has focused on the neutrophilic response to this filamentous form of the microbe. As Candida destruction necessitates a full-blown response to both forms of the organism, work in our lab has focused on this gap in our understanding of anti-fungal host defense. We found that *C. albicans* hyphae growing in the kidney of an infected rat induced massive clustering of inflammatory neutrophils that entirely surrounded the hyphae (11). This clustering of human neutrophils could be replicated *in vitro* with *C. albicans* hyphae plated on fibronectin but not on hyphae plated in the absence of fibronectin (13). Pretreating hyphae with an anti-β-glucan antibody prevented clustering of neutrophils suggesting that the β-glucan component of the fungal cell wall is important for neutrophil responsiveness (13). Immobilization of purified β-glucan in the presence of fibronectin was a biomimic for Candida hyphae within tissue ECM suggesting that fungal β-glucan is necessary and sufficient for homotypic aggregation (13). Furthermore, this swarming and aggregation took place rapidly, being evident in less than 30 min *in vitro* (13).

## Adding NETosis to the Repertoire of Neutrophil-Mediated Immunity

NETosis was initially described as a pathway of chromatin decondensation and release with requisite NADPH oxidase, elastase, and myeloperoxidase activity in response to activating stimuli (54–56). The initial reports showed relatively slow kinetics, occurring hours following exposure to stimuli, including bacteria, fungi, or PMA (14, 17, 54, 55, 57, 58), though evidence suggests that no *de novo* gene synthesis is required (59). As additional investigators explored conditions necessary and sufficient to NET release within their experimental systems, some variance in the original paradigm emerged. The “classical” pathway involves entry of the neutrophil into a cell death program that requires ROS and manifests in plasma membrane disruption and NET release 1–4 h after stimulation. This pathway utilized peptidyl arginine deiminase type IV (PAD4) for histone citrullination that leads to chromatin decondensation due to neutralization of histone electrostatic charge normally imparted by arginine but lost upon conversion to citrulline (60). Elastase and myeloperoxidase serve to digest nuclear histones after translocation such that absence of these enzymes impairs NET release (61). A more recently identified early/rapid, or “vital,” NET release was identified that can result in extrusion in minutes, independently of ROS and without compromising cell viability, in response to *Staphylococcus aureus, C. albicans*, and Leishmania promastigotes (13, 62, 63). The “classical” and “vital” NETosis pathways need not be mutually exclusive, as the context of NETotic stimuli presentation, such as timing, viability, size, or morphotype, can drive differential response patterns and kinetics (13, 14, 54, 57, 62–64). ECM ligands in the context of tissue infection can also drive differential neutrophil responses.

## Role of Integrins and Extracellular Matrix in the Regulation of NETosis

Our laboratory has demonstrated an integrin-dependent ECM response that both actively suppresses the respiratory burst to Candida hyphae, or immobilized fungal β-glucan, while driving a robust, rapid NETotic response (12, 13). Additionally, work with neonatal neutrophils show that this NETotic anti-fungal pathway is active even though neonatal neutrophils have been shown to be deficient in NETotic responses to other initiating agents, underscoring the importance of stimuli context in evaluating effector function (65, 66).

In addition to our work, evidence to date describes the role of β2 integrins in NET release as it occurs along liver sinusoids or vascular endothelium. Platelet–neutrophil interactions have been shown to occur under conditions of severe sepsis (67) or endotoxemia in which activation of platelet TLR4 promotes platelet binding to neutrophils with ensuing NET release (68). Two recent studies differ with regard to which β2 integrin mediates platelet–neutrophil binding. McDonald et al. (69) show a role for LFA1, although the ligand on the platelet remains to be defined. Rossaint et al. (70) showed that incubation of stimulated platelets with neutrophils *ex vivo* induced NETosis that could be blocked with anti-CR3 antibodies but not with antibodies against LFA1. Mohanty et al. (71) recently identified that neutrophils form NETs from saliva exposure in a β2 integrin-independent fashion, as LAD1 patients form NETs to saliva and PMA but not to unopsonized *S. aureus*.

Complement receptor-3 has been shown to regulate apoptosis of neutrophils such that the genetic absence of CR3 delayed the onset of apoptosis of neutrophils after thioglycollate injection (72). Given that CR3 determines a NETotic pathway for Candida, it is of interest to consider whether or not NETotic and apoptotic pathways have common points of regulation. Evidence to date suggests that NETosis and apoptosis both require calcium for initiation but then show divergence in the sense that PAD4 activation does not depend on downstream components of the apoptotic pathway, such as activated caspase, and apoptosis does not depend on PAD4 (73). Indeed, histone citrullination in neutrophils is induced by inflammatory stimuli and not by treatments that induce apoptosis (73). Moreover, treatment of neutrophil-differentiated HL60 cells with calcium ionophore showed that histone citrullination preceded PARP cleavage, such that the decision-making events may be temporal.

The occupancy of one integrin by ligand has been shown to be capable of suppressing the function of other integrins in a phenomenon referred to as trans-dominant inhibition, or integrin crosstalk. For example, activating antibodies specific for the αvβ3 integrin suppress α5β1-dependent phagocytosis and ligation of α4β1 inhibits α5β1-dependent expression of metalloproteinases (74, 75). Ligation of αIIbβ3 induces trans-dominant suppression of target integrins α5β1 and α2β1 (76). Additionally, antibody activation of β1 integrins was shown to increase CR3 adhesion to fibronectin (77) and outside-in activation of β2 integrins via crosslinking was demonstrated to upregulate the expression of β1 integrins (78). These studies suggest that certain integrin-specific ligands provoke integrin crosstalk that could result in alterations in cell migration and invasion. With regard to the anti-Candida response of human neutrophils, we discovered a temporal, interregulatory relationship between the β2 integrin CR3 and regulation of β1 family members and this modulates the response to β-glucan or *C. albicans* hyphae in the context of ECM (12, 13, 49–52). The extent to which integrin crosstalk operates as a regulatory pathway for other innate immune functions is not well understood.

## Neutrophils, Integrins, Extracellular Matrix, NETosis, and Beyond

The host response of neutrophils to *C. albicans*-infected tissues necessitates ECM contact. Our work and others have clearly demonstrated a regulatory role of ECM in determining neutrophil function, including NETosis. The focus of this review, the role of ECM ligation by neutrophil integrins as it pertains to both host defense functions and the kinetics of these functions, has implications that reach far beyond the anti-fungal response. The totality of ECM involvement in neutrophil host defense in tissues makes accounting for both its presence and the role of integrin engagement an important and under-examined mechanistic aspect of inflammation.

## Author Contributions

XO and JR conceived, designed, and wrote the manuscript.

## Conflict of Interest Statement

The authors declare that the research was conducted in the absence of any commercial or financial relationships that could be construed as a potential conflict of interest.

## Abbreviations

CARD9, caspase recruitment domain-containing protein 9; CR3, complement receptor 3; ECM, extracellular matrix; LAD, leukocyte adhesion deficiency; NETs, neutrophil extracellular traps; PAD4, peptidyl arginine deiminase type IV; PAMP, pathogen-associated molecular pattern; PRR, pattern recognition receptor; ROS, reactive oxygen species.
